# Exhaustion disorder: the genesis of a diagnosis that exists only in Sweden

**DOI:** 10.1007/s11019-025-10292-5

**Published:** 2025-08-19

**Authors:** Fredrik Svenaeus

**Affiliations:** Södertörn University, Stockholm, Sweden

**Keywords:** Exhaustion disorder, Burnout, Stress disorders, Sickness leave, Life narrative, Phenomenology of illness, Culture-bound syndromes

## Abstract

In a scientific report published 2003 a psychiatric research group in Sweden proposed to the National Board of Health and Welfare that a new diagnosis with the name “exhaustion disorder” (“utmattningssyndrom”) (ED) should be created in the Swedish diagnostic system. Two years later the board approved the proposal and the diagnosis was registred in the Swedish version of ICD-10. Since 2005 the prevalence of ED in Sweden has gradually increased and at the current date more than 40 000 people are on long-term sick leave as a result of the diagnosis. Interestingly, there is no corresponding medical diagnosis outside of Sweden, although patients in other countries are declared ill with similar symptoms, receiving other diagnoses, such as burnout, depression, acute stress or adjustment disorder. In this paper, the history of ED is told and an attempt is made to answer the question why it has come to exist and prevail in Sweden despite no evidence of validity. The analysis is performed by scrutinizing the criteria for the diagnosis and how it has been connected to the granting of sick leave in the Swedish social insurance system. In conjunction with this, a phenomenological analysis is provided of how ED has been named and interpreted in the Swedish society as a particular form of life-narrative pattern. This pattern of break-down and rebuild of a more in-tune-with-nature version of oneself in recovering from ED is found in Swedish popular culture, and it is supported by academic studies, self-help books and the strategies of rehabcenters.

## Introduction

In this paper, the history of exhaustion disorder (“utmattningssyndrom”) (ED) is told and an attempt is made to answer the question why the diagnosis has come to exist and prevail in Sweden, despite the lack of evidence for it forming a distinct category in comparison with other psychiatric disorders. The diagnosis in question is only used in Sweden and was introduced in WHO: s diagnostic system ICD-10-SE in 2005 by The National Board of Health and Welfare with the code F43.8 A (Socialstyrelsen 2017). ED is standardly meant to be identified *instead* of depression, anxiety or other psychiatric disorders when diagnosing patients with symptoms caused by chronic stress in working life (comorbidity is still an option in some cases). By introducing ED in ICD Sweden made the choice of not making use of the internationally well-known concept of burnout (I will return to this choice in more detail below).

The investigation of ED will be performed by scrutinizing the criteria for the diagnosis and how it has been connected to the granting of sick leave in the Swedish social insurance system. In addition to this, a phenomenological analysis is provided of how ED has been constituted and interpreted in the Swedish society as a particular form of life-narrative pattern present in radio programs and novels, supported by academic studies, self-help books and the strategies of rehabcenters. Is ED a real disorder or rather a Swedish anomaly that will soon be abandoned? Or will rather other countries follow Sweden in identifying this peculiar psychiatric condition as a major cause of disability and suffering? In the paper, I will aim to provide the history of ED in Sweden, explain the success of the diagnosis by way of a phenomenological analysis of illness and other forms of human suffering, and point to some challenges for the future. The details of the history of ED are important since they show that the coming into existence and success of a medical diagnosis can be the result of many different powers at play: changes of life conditions, changes in health-insurance regulations, proposals made by influential researchers, and, last but not least: changed labels and concepts in the heads of doctors and patients, thematized by way of podcasts, novels and social media.

The most important scholar who has pointed to and analyzed this *interactive* nature of psychiatric disorders is probably Ian Hacking, and the reflections below are inspired by what he did achieve in books such as: *Rewriting the soul: Multiple personality and the sciences of memory* (Hacking 1995), and *Mad travelers: Reflections on the reality of transient mental illnesses* (Hacking 1998). According to Hacking, medical diagnoses, and particularly so in the case of psychiatric disorders, can be natural – residing in the brain – and culturally interactive at the same time, since patients become aware of and are affected by the labels they are classified with – such as the label of ED. As we will see, a phenomenological approach to psychiatric disorders supports and further develops such a view by stressing the bodily as well as culturally dependent nature of human suffering.

## What kind of creature is a psychiatric disorder?

Before telling and analyzing the history of ED in Sweden some basic things about the ontology of mental disorders and the establishing of psychiatric diagnoses are in place. A mental disorder or syndrome (the words can be used interchangeably) in contrast to most somatic diseases are not established by way of any bodily examination or biological testing. Instead, mental disorders are diagnosed by way of identifying and grouping key symptoms and clinical signs experienced and retold by the patient in the clinical encounter. The psychiatrist (or other physician) makes use of her/his clinical experience and of criteria checklists found in diagnostic manuals when s(he) makes her/his diagnostic judgement. Nevertheless, the main idea in psychiatry is that diagnoses represent *dysfunctions*, eventually to be found in the patient’s brain, one way or the other:

A mental disorder is a syndrome characterized by clinically significant disturbance in an individual’s cognition, emotion regulation, or behavior that reflects a dysfunction in the psychological, biological, or developmental processes underlying mental functioning. Mental disorders are usually associated with significant distress or disability in social, occupational, or other important activities. (APA 2013, p. 20)

This quote is from the introduction of DSM-5, the most important diagnostic manual in psychiatry, and compatible with ICD-11, the manual developed by WHO and which is being used in Sweden. It has been pointed out by many researchers and philosophers that DSM and ICD do not really live up to this definition of mental disorder, since the criteria lists of the many different psychiatric disorders present in the manuals (around 300 in DSM-5) do not identify, but merely *assume*, the presence of some kind of dysfunction in the patient (Horwitz 2002). The argument seems to be that the identified “distress or disability” – the painful symptoms and deviant behaviors – by themselves indicate “a dysfunction in the psychological, biological, or developmental processes underlying mental functioning”. However, this conclusion is highly debatable, since there appears to be no way of drawing a sharp line between psychiatric disorders, on the one hand, and human distress and behaviors that are painful and culturally deviant but nevertheless do not reflect medical dysfunctionality, on the other.

The developers of the diagnostic manuals have certainly tried to guard themselves from this type of objections and the quote above therefore continues:

An expectable or culturally approved response to a common stressor or loss, such as the death of a loved one, is not a mental disorder. Socially deviant behavior (e.g., political, religious, or sexual) and conflicts that are primarily between the individual and society are not mental disorders unless the deviance or conflict results from a dysfunction in the individual, as described above. (APA 2013, p. 20)

Unfortunately, there is still no scientific way to identify dysfunction in the individual and the medicalization charge is therefore hard to fend off (Frances 2013; Paris and Phillips 2013).

A phenomenological approach to mental disorders would in contrast to the DSM-approach stress that psychiatry is dealing with illness(es) rather than brain dysfunctions, that is: human experiences of suffering corresponding to various individuals ways of inhabiting their everyday worlds (Heidegger 2001). Along these lines, as developed by the present author in *Phenomenlogical Bioethics*:

Suffering is an alienating mood overcoming a person and engaging her in an embodied struggle to remain at home in the face of the loss of meaning and purpose in life. Such a mood (or combination of moods) involves painful experiences at different levels of the person’s being-in-the-world that are connected but are nevertheless distinguishable by being primarily about, firstly, my embodiment, secondly, my engagements in the world together with others, and, thirdly, my core life-narrative values. (Svenaeus 2018, p. 36)

Somatic illness typically involves existential suffering only in severe cases, whereas psychiatric illness (disorder) involves existential suffering in most cases, including the cases referred to as mild and looked upon as bordering to unhappiness (e.g. mild cases of depression). The bodily aspects of psychiatric illness become more pronounced and visible in severe cases, when the mood is alienating to the point of making the body heavy (depression) or strange (psychosis) (Ratcliffe 2008, 2015).

That existential suffering is typically involved in a different and more constitutive way in mental disorder than in somatic illness means that psychiatry is even more prone to medicalizing tendencies than somatic diagnostics (Rose 2019). What is often brought in focus as the down side of medicalization in psychiatry is the increased prescription pharmaceuticals, such as antidepressants or ADHD-medication, but an even greater danger from the phenomenological viewpoint lies in the standardization brought by the increasing use of diagnostic manuals. The risk with using diagnostic manuals in psychiatry is not only over-diagnosis and the medicalization of healthy behaviours and feelings (which are both cases of everyday or existential suffering rather than illness by the phenomenological characterizations given above). The risk is also that persons are stereotyped, made into their diagnoses instead of being approached and understood in an empathic and hermeneutic manner, addressing life-world concerns, that is characteristic of good health care (Svenaeus 2018, pp. 55–74). The effects of Ritalin may have a stereotyping effect as concerns moods and ways of being-in-the-world, but even more so does the tendency to map and sort persons in diagnostic categories like ADHD.

Diagnosis is a necessary and important instrument of medical practice, but when it is used to categorize human experiences and behaviours rather than to explain what has gone amiss in the body, it blocks the view of what should be the real concern of the doctor and health care team: the patient’s being-in-the-world (Heidegger 2001). DSM wants to create the impression that doctors can explain and cure the sufferings of the soul in the same manner as they explain and cure the sufferings of the body, but, of course, they cannot, since life-world matters and existential questions are not amenable to biological analysis in the way the functions of the body are. Diseases can be detected with the help of medical technology, and the way they cause illness suffering can be explained by medical science. Mental disorders can at best be described in everyday language and understood by way of good psychiatric practice.

The reason for these differences between mental and somatic illness is not only that the brain is the most complex organ of the body; it is also that existential and everyday sufferings are enmeshed in psychiatric disorders in a manner that often makes it hard to discern what is the most important *reason* for the suffering in question. Did the poverty and family-related problems of a person lead to depression, or was it the other way around? No brain scans can answer this question; only the life story of the patient told in a meeting with a psychiatrist (or other health care professional) can. From the phenomenological point of view, the difference between illness suffering and other forms of human suffering cannot be defined by way of brain-disease findings, even though abnormalities in biological functions of the body (including the brain) could be taken to *indicate* that the suffering is medical in nature. However, when it comes to the functions of the brain, the challenge of defining normal intervals is even harder than in the cases of somatic illness, inevitably leading us back to the *experiences* of potentially diseased/disordered people (Aho and Aho 2008; Carel 2016).

The problem of establishing a sharp line between mental disorder, on the one hand, and distress due to aversive life events or socially deviant behavior, on the other, is relevant for all psychiatric diagnoses, but more or less so with different disorders and in individual cases. The problem becomes especially pertinent when we are discussing *contested diagnoses*, that is: diseases and disorders which are openly challenged by researchers and the public to not be “real” in the sense that they do not correspond to any existing dysfunction in the body/brain of its bearer (Schone 2020). In such cases it is not only some of the diagnoses being made that are challenged but the medical condition as such. Well known examples of such contested diagnoses are chronic pain conditions, such as fibromyalgia, and chronic fatigue conditions, such as ME (Myalgic Encephalomyelitis), or post covid.

Exhaustion disorder could certainly be considered as such a contested illness and this needs to be taken into account when examining its short history. In accordance with this pattern of contestation, persons who are diagnosed with ED are not generally considered to be malingering but it is nevertheless often contested that the condition they are suffering from is a real illness and not just ordinarylife-related exhaustion in a more general sense. It should also be noted that there are other, much older, diagnoses in the history of psychiatry that display a similar symptomatology in comparison with ED. The most striking and famous example is probably neurasthenia (Shorter 2013).

That ED is only diagnosed in Sweden makes the medicalization charge – i.e. that ED is not a real illness – even easier to press, but it should be noted that the world of psychiatry includes several culture-bound syndromes that are nevertheless accepted and listed in diagnostic manuals, and have since long been studied by social anthropologists (Kirmayer et al. 2014; Kleinman 1980). Just to mention one example, in Japan *taijin kyofusho* is regarded as a specific type of mental disorder that includes a fear of embarrasing others by way of one’s inferial appearance and even (imagined) foul body odour.^1^ Such culture-bound syndromes in psychiatry often refer to patterns and beliefs consonant with animistic and religious world views, but, as we will see with the Swedish example of ED, psychiatric labels can also interact with secular cultural patterns and more pragmatic concerns having to do with the labour market. In order to show this I will now turn to a very recent history of economic crisis, politics of sick leave rules and new cultural ideals in Sweden.

## Exhaustion in Sweden since the 1990s

In 1992, a severe economic crisis hit Sweden, and the job market went through a gradual streamlining process with high unemployment and increased pressure on the remaining work force to do things faster and more effectively as a result. This was particularly visible in health- child- and elderly care, and in the school system, sectors funded by the state and municipalities by way of tax money. The Swedish government and the municipalities made major cut downs in the social security and pension system, but also in the funding of schools, hospitals and other welfare institutions. The private sector went through a similar streamlining process because of the economic crisis. This is arguably what started the epidemic of what was then known as “utbrändhet” (burnout) in caring and teaching professions in Sweden, which has led to the high prevalence of ED in the population that we see today. Stressful conditions of working life as a result of economic cut downs are certainly not unique to Sweden. Many other countries suffered depression in either the 1990s or the 2000s and went through a streamlining of the job market as a result. However, nowhere else was a new psychiatric disorder put in place to answer up to the surge of chronic stress experienced by the work force.

In 1997 sickness leave in Sweden began to rise dramatically, a development that peaked in 2002 when the average number of days on sick leave in the country increased by almost 100%, reaching 18 days a year (Lytsy et al. 2009).^2^ The escalating numbers were most probably the delayed effect of the 1992 crisis with a slowly recovering economy (people do not call in sick when still unemployed or when struggling to keep their position). In 2003 new regulations of the Swedish health insurance system were put in place by the politicians to stop this development, and the sick leave days began to fall steeply, reaching an all-time low in 2010 of 6 days a year (ISF 2023). The number of average sick leave days then rose again, reaching 10 days in 2017, a number which has been preserved until the present day. Not counting the cases of covid-19 and post covid, this rise, and high-level plateau, are largely a consequence of a new diagnosis which has produced a large number of cases of long-term sick leave: “utmattningssyndrom” (exhaustion disorder) (Försäkringskassan 2023).

Counting the number of cases and days of sick leave the diagnosis of ED has undoubtedly been a huge “success”. In 2023 more than 40 000 persons were on sick leave for ED in Sweden and the average time of recovery was more than six months (Försäkringskassan 2023). 30 000 of these patients are women and most of them are in their 30–40s. Sweden has a population of 10,5 million people and about 6 millions of these are in working age, which makes it possible for them to be on sick leave. About half a million of the six million people are unemployed and another half a million are students, so this means that the prevalence of ED in Sweden is about 0.8%. However, the prevalence would probably end up much higher if persons currently unemployed or on disability leave were included in the calculation.^3^

In 2003, after having performed an exploratory interview study with patients, a psychiatric research group in Sweden, led by professor Marie Åsberg, proposed in a report to the National Board of Health and Welfare (Socialstyrelsen) that a new diagnosis with the name “exhaustion disorder” (“utmattningssyndrom”) should be added in the Swedish diagnostic system (Åsberg 2003).^4^ The National Board of Health and Welfare approved the proposal, and in 2005 the diagnosis was created in the Swedish version of ICD-10 with the code F43.8 A. Three reasons are given in the report for introducing the new diagnosis. The first reason is to avoid the detrimental associations of “having been burnt out” like a machine, or similar created by the diagnostic concept of burnout, which is actually not conceived of in ICD-10 as a medical disorder but as a “problem associated with employment or unemployment”. The second reason is to avoid stigmatizing patients by making use of the diagnosis of “adjustment disorder” found in ICD-10, since this disorder by logic of the label tends to put the blame on the individual for having difficulties to adjust to changed life circumstances. Lastly, the third reason is to avoid mixing up patients suffering from ED with patients suffering from depressive disorders, since the patients, according to the authors of the report, belong to separate, admittedly sometimes overlapping, groups.

In the report from 2003 the new suggested (and in 2005 accepted) diagnostic criteria for ED were listed as follows:

(A) Physical and mental symptoms of exhaustion for at least 2 weeks. The symptoms have developed in response to one or more identifiable stressors, which have been present for at least 6 months.
(B) Markedly reduced mental energy, manifested by reduced initiative, lack of endurance or increase in time needed for recovery after mental efforts.
(C) At least four of the following symptoms have been present most of the day, nearly every day, during the same 2-week period:

1. persistent complaints of impaired memory and concentration.
2. Markedly reduced capacity to tolerate demands or to perform under time pressure.
3. Emotional instability or irritability.
4. Insomnia or hypersomnia.
5. Persistent complaints of physical fatigue and lack of endurance.
6. Physical symptoms such as muscular pain, chest pain, palpitations, gastrointestinal problems, vertigo or increased sensitivity to sounds.

D. The symptoms cause clinically significant distress or impairment in social, occupational or other important areas of functioning.
E. The symptoms are not due to the direct physiological effects of a substance (e.g. misuse of a drug or medication) or a general medical condition (e.g. hypothyroidism, diabetes, infectious disease).
F. The stress-related disorder does not meet criteria for major depressive disorder, dysthymic disorder or generalised anxiety disorder. [If, for example, criteria for depression are met, exhaustion disorder is to be used as a complementary diagnosis.] (Lindsäter 2022)

As is clear from these criteria, which were adopted in ICD-10-SE as F43-8 A, the differential diagnostics is to be carried out with particularly depressive disorders and anxiety disorders in view. The report behind the diagnosis also makes clear that the “identifiable stressors, which have been present for at least 6 months” are mainly thought to be found in working life. The authors of the report from 2003 are concerned that an increasing number of patients with exhaustion symptoms are being diagnosed as depressed even though the symptoms are clearly caused by working life stress, in contrast to other stressors or endogenic processes thought to be responsible for depression and anxiety disorders. The label of “exhaustion depression” (“utmattningsdepression”) had been used more or less informally by Swedish doctors (and the public) since the 1990s, but the authors of the report think it is time to create a new diagnostic label which is not to be perceived as a subspecies of depression, but as an entirely different sort of psychiatric disorder altogether, belonging to the “Reaction to severe stress, and adjustment disorder category” realm (F43 in ICD-10).

As explained above, the launch of the new diagnosis in 2005 coincided with new regulations of health insurance in Sweden, making it harder to be granted sick leave if not fully incapable of carrying out the type of activities necessary for performing one’s job. The new rules that were put in place in 2003 also put a cap of the number of days a person may be on sick leave without applying for other jobs more suited to the person’s restricted capabilities. All in all, it became much harder to be granted long term sick leave in Sweden, not primarily because doctors had changed their diagnostic behaviour, but because The Swedish Social Insurance Company (Försäkringskassan) had been given a new role as a gatekeeper of the Swedish health insurance by the politicians (Hultgren and Barmark 2008). The reason for the new rules put in place 2003 was the exploding numbers of patients on never-ending sick leave with health problems caused by chronic stress, which had led to a huge public debate in the country. The discussion in Swedish media about malingering individuals faking illness to get out of boring jobs was the most visible part of this debate, but other important issues were also non-successful rehabilitation programs and over-demanding jobs.

To sum up, although doctors in Sweden in 2005 were offered a new diagnostic possibility in encountering patients with symptoms caused by chronic stress in working life, the opportunities to grant them full- or long-time sick leave were simultaneously being hampered by new sick leave rules and the gate keeping role performed by the administrative personnel at Försäkringskassan. This all began to change in 2008 when politicians responded to the criticism levelled against Försäkringskassan expressed by the many persons who had ended up in desperate life situations as a consequence of rejection of sick leave despite being unable to work (ISF 2023, pp. 39–42). As a result of this, the rules and instructions to Försäkringskassan made by the politicians were changed again in 2009, recommending a minimum of 6 months of sick leave to allow sufficient recovery if a patient was diagnosed with ED, and as a consequence of this change the number of days on sick leave started to move upwards again in 2010.

Presently, the number of persons on long term sick leave in Sweden is still rising and the majority of the patients in this group have been diagnosed with psychiatric disorders, particularly with ED or other stress related conditions (Försäkringskassan 2023). The critique of the judgements made by Försäkringskassan and the difficulties to be granted long term sick leave is still very much on the public agenda until this day, and many of the cases that have surfaced in the media since 2005 are precisely cases of ED (Altermark 2020). Other diagnoses responsible for long term sick leave in Sweden are Chronic Fatigue Syndrome/ME and post covid, which are diagnosed by way of similar symptoms as those present in ED but allegedly caused by long term effects of virus infections.

## Cultural images of exhaustion disorder

The history of ED in Sweden is not only a history of diagnostic codes and health insurance regulations. It is also a cultural history in which everyday descriptions of ED and burnout conditions, “utbrändhet,” have entered the minds of Swedes in a very powerful manner. ED has been established as a form of illness with a peculiar pattern that maps on to all three levels of the phenomenological analysis of suffering developed above: alienated feelings of embodiment, unhomelike being-in-the-world, and challenged identity of the afflicted person (Svenaeus 2018). “Utbrändhet” (the Swedish translation of burnout) has become the everyday expression for ED and similar less severe conditions. A search in the national library system Libris on “utmattningssyndrom” (ED) generates no less than 664 books in Swedish (Libris 2025).^5^ This is a rather remarkable number, considering the fact that we are dealing with a diagnosis with such a short history.

Some of these books are novels, most of them are biographies or self-help books, and some are dissertations, research reports and books being used in the education programs of psychiatrist and psychotherapists. There are also children books trying to explain to children why their parents or teachers have become ill by the circumstances of working life situations and online stress (Backe and Ingvarsson 2022). There are even books about children and adolescence experiencing burnout themselves because of a stressful home- or school environment (Andersson 2023). In addition to the 664 books, there has also been thousands of articles published in newspapers and magazines, often with detailed stories of/from persons falling ill with ED and/or “utbrändhet” (burnout), not to mention the countless blogs and webpages devoted to the syndrome.

A particularly influential form of cultural image of ED has been provided by Sveriges Radio (public radio in Sweden) by way of the well-established radio program series “Sommar”, which is broadcasted every day during roughly two months in summertime in Sweden (Sommar & Vinter i P1. 2025). Each day a famous Swede – athletes, authors, artists, actors, directors, entrepreneurs, journalists, philosophers, politicians, researchers and other influential and well-known individuals – are offered one and a half hour of premium broad cast time (1-2.30 pm) to tell their life stories and/or inform the public about important subjects of their expertise. Many Swedes listen to some of these programs during their summer holidays and discuss them within their family or circle of friends. The program format has been in place since 1959 and “Sommar” is also available as a pod cast series since the early 2000s, to be streamed and listened to at preferred time.

Interestingly, the last 15 years or so, a peculiar narrative pattern has become common in the life stories told by the program hosts of “Sommar”.^6^ It is a story of hard labour and often success followed by a breakdown labelled precisely as burnout or ED. The price to be paid for working too much and not taking proper care of oneself is a total and brutal exhaustion kicking in suddenly, very much described by way of the symptom types of A, B, C and D in the criteria given above. After having described this dramatic break down, the program host tells the story of a long way back to health and self-confidence from the misery of ED. By way of strenuous rehabilitation efforts – assisted by professionals, friends or entirely on their own – they have learned what really matters in life: to take proper care of yourself and your close ones, and to do things that you really find meaningful in life, not because they bring you fame and money, but because they are valuable in themselves and makes the world a better place to live in. Certainly, such stories are not unique to Swedes or Sweden, but the presence of ED has probably made them even more frequent and compelling.

Ironically, the journey back from burnout is most often what has made the program hosts of “Sommar” even more successful and well known – otherwise they would not have been invited to do the program – and sometimes they have even made their fame by writing a book about ED after the comeback. This comeback-story narrative-pattern is somewhat novel in comparison with most other comeback-stories that have been told since the dawn of history. The reason for defeat and break down is not an opponent or oppressor, or some evil state or organization, but the person *herself*, who has lived in a reckless way, not guarding herself from the dangers of harmful stress. There are some similarities with comeback stories being told about drug addiction, and sometimes these two types of stories can be brought together in one “Sommar” program, if the lifestyle of the hard-working person has also included partying and drinking. Most often, however, the hard work or fame-seeking behaviour in itself is considered to be the drug that needs to be weaned off to return to a healthy life.

Of course, most people being diagnosed with ED never achieve any fame in the way the program hosts of “Sommar” have done, but the story pattern itself helps also non-famous listeners to make sense of their sufferings and imagine how their misery could be eased. In many cases, cultural images of ED and burnout situations that are provided in the media, and talked about in the workplace or at home, make people think about their entire life situation in a new way. This includes all three levels of suffering as an alienated mood, identified by way of the phenomenological layout: body, world and personal identity (Svenaeus 2018). The programs also make the listeners suggest the diagnosis to their doctors, who may also, by the way, have been listening to “Sommar”. In this way such stories are highly contagious.

A particular subspecies of the narrative plot of ED is told by the woman who has been taking care of others – family and friends – instead of attending to her own needs. This she has done while simultaneously making a career – ending up in an influential position – and such stories allows for a blend-in of political equality claims. Three quarters of the patients being diagnosed with ED are women and the probable explanation for this is that women take care of the larger share of household work in addition to working full time in Sweden (Försäkringskassan 2023). Another reason why women are overrepresented is probably that they in addition to a heavy workload spend more time on social media than men do (Haidt 2024). Quite a few of the books that have been written since the early 2000s about ED have titles with words like “good girls” and “accomplishment princesses” bringing a feminist analysis to the subject (Ernsjöö Rappe and Sjögren 2004; Rose and Perski 2008; Tengblad 2017). There are also many books about ED and the caring professions, making the link between cut downs that have made doctors, nurses and other care workers experiencing “ethical stress” and “burn out fatigue” leading to ED. Women are over-represented in such professions, and this partly explains why three out of four persons with the diagnosis are women.

## Healing stress and exhaustion by way of green rehab: two recent examples

Two recent examples of Swedish books that tell the story of falling ill and recovering from ED are Annika Norlin’s *Stacken* (“The Ant Hill”) (2023) and Veronica Linarfve’s *Bidrottningen* (“The Queen Bee”) (2024). Both books are novels, but they have found inspiration in self-experienced ED and their stories, particularly in the latter case, display many similarities with their respective biographies (Linarfve 2024, p. 286). The two books do not only illustrate the typical narrative pattern of ED identified above: a dramatic break down and a long struggle back to health and self-confidence by way of which the afflicted person learns what really matters. They also do so by way of prescribing a particular rehabilitation program: attempting to get closer to nature, living more like an innocent and confident animal than as a stressed out and confused human being,. The books also bring out the examples of two particular animals to merge with which are both species of insects: ants and bees. This could be considered as a particularly potent form of bodily alienation, stretching from the lived bodily pattern of experiencing the whole world, all the way to the cultural critique of the human life form as a being out of tune with nature itself in our contemporary predicament.

Let us begin with the bees (Linarfve 2024). Gabriella, in *Bidrottningen*, exhibits all the typical risk behaviors of a middle-aged woman ending up with ED. Gabriella spends all her time trying to keep her demanding boss, absent husband and spoiled children happy by carrying out every task needed for a smooth and successful work- and family life. She plans everything meticulously 24/7, assuming full responsibility for the social and administrative matters at the advertising agency where she works and handling all the infra-structure issues of family life, even writing the homework tasks for her two children aged eight and ten. She also takes care of her grandfather who lives in a separate house with beehives in the garden. Gabriella writes lists in spread sheet computer programs and arranges systems with post-it notes on the walls in the kitchen. She worries for each and everybody, including not only her own family and relatives but also her friends and working mates. She sleeps less and less, having a nagging sense of always having forgotten something very important, which she cannot remember any more (in the didactic ending of the novel this something turns out to be herself, of course).

Gabriella’s breakdown is a classic illustration of ED symptoms, though hers are somewhat extreme: she arrives at her former workplace in a state of confusion and panic, refusing to leave her old desk even after being told she no longer works there. Eventually, the police and a medical team are called when she threatens to jump out of a window and hides under the desk, unwilling to speak or move. What makes Gabriella’s case unique is the psychotic dimension of her symptoms, which the reader experiences through her altered perceptions and behaviors. She believes she is transforming into a bee, with extra legs and wings emerging from her body, and begins to see, hear, and smell the world as if through a bee’s senses. She is subsequently taken to a psychiatric clinic, where she is sedated and observed, then later treated with additional medications and psychotherapy.

In the long process of recovery, Gabriella not only identifies with a bee, but also spends a lot of time in the garden of her now deceased grandfather’s house, where she learns to tend to the plants and the beehives. She has been told as a child that she is allergic to bee stings, but this turns out not to be true, and through moving closer to the plants and the animals, and by keeping a distance to her family and working life, she finds peace and new strength in life, not through turning into a bee – this remains a tempting but also scarry alternative – but through being *with* the bees and *in* the garden.

Annika Norlin’s *Stacken* is more of an experimental and intellectual approach to ED than the straightforward didactic example of the queen bee in *Bidrottningen* (Norlin 2023). The story begins with the breakdown story of a young woman on temporary and freelance jobs, trying to prove herself worthy of a permanent position as a journalist, and with a number of shallow acquaintances that she entertains during free evening and nights, including a couple of sexual partners. She has bought herself an expensive apartment in a big city and never leaves town except for jobs, trying to live up to the standards of an independent and successful career woman, socializing with her peers in bars, fitness studios and concert halls.

One morning Emelie – the main character of the novel – is unable to get up from her bed. She feels paralyzed and exhausted, her heart is beating too fast, and she is having panic attacks. After having been saved by her neighbour, who has a spare key for her door, she is taken to a health-care centre and diagnosed with ED in the standardized way of applying the symptom list we explored above:

- How do you feel now?
- I, I do not know.
- Here is a form that I want you to fill out with different symptoms to be estimated.

(Pause.)

- You are not filling out the form.
- No, I, I am unable to do so. Excuse me. What did you say?
- Emelie, I can read you the questions instead. Does that sound good to you?
- Yes, that sounds… good. (Norlin 2023, p. 14, my translation)

Emelie’s neighbour is an older Sápmi woman – the only exception to her otherwise standardized social pattern of career people – who takes her outside the city for a walk in the forest. Emelie has never been fond of spending time in nature but the contrast with her hectic and shallow city life is truly striking to her. After having cut loose from her previous colleagues and acquaintances Emelie decides to go further out in the countryside and live in a tent in the woods. One day she sees from a distance how a strange bunch of people gathers by a lake, three women, three men and a child. They make a fire, sing incomprehensible songs and then go swimming naked in the lake together. They prepare a bird and grill it with pieces of dough on the fire and then say “thank you” to each other and to the bird before they start to eat. Lastly, the lie down closely together, not in a sexual manner, but rather striving to form one single body, keeping themselves warm and preserving unity in front of the fire.

Emelie gets to know the seven persons of the group and it turns out they are attempting a commune living disconnected from society in an old house by the end of a small road. They are trying to live from what nature provides them with – farming and hunting – spending most of their time in nature not reflecting upon things but living by instinct in the way of animals. They name themselves “The Ant Hill” – which is also the name of the novel – and by this they intend that everyone has a separate function which is necessary to the survival and flourishing of the commune – the way ants divide the working tasks of the ant community in-between themselves. The seven persons form one being, but there turns out to be an ant queen, that is the informal leader of the group, as well as ant workers and ant soldiers with specialized knowledges and capacities depending upon their skills and interests.

Norlin’s novel is not idealizing nature and prescribing a recipe for ED by way of wildlife (or garden life) recreation in the same straightforward manner that Linarfve does in her book. The ant hill commune turns out to hide deep and dirty secrets, and nature is not only portrayed as a paradigm for a more healthy and happy human life in the novel, but also as a cruel reality of anger, fear and domination. But the idea of living by way of feeling and instinct in a small group that unburdens the individual from shouldering personal responsibility nevertheless materialises as a blueprint for escaping contemporary working- and family life in the city. Western lifestyle and the destroying of natural eco systems is criticised in the book in a similar manner to what is found in the novel by Linarfve, in which the dying out of bee communities is a particular issue. In Norlin’s novel, the old trees of the natural forest are the main focus, a habitat that is being replaced by modern forest management. During summertime, the commune sleeps together under the oldest tree found in the forest – “Stora gran” – lying down close together protected by the big branches.

To sum up, these two novels exemplify and concretize the recurring narrative pattern of women experiencing and recovering from ED discussed above. The different levels of suffering – embodied, enworlded and personal-identity bound – are presented as alienating life patterns to be coped with through changed life style and a political critique. However, rather than focusing on feminist critiques of patriarchal structures, they present an ecological criticism of Western lifestyles and capitalism. ED is portrayed as a consequence of living disconnected from nature and one’s bodily rhythms, becoming infected by artificial values of modern urban life and technical gadgets such as smart phones. Women, in particular, appear more susceptible to develop ED symptoms, yet they also possess unique pathways to reconnect with nature through their embodied experiences. In this way, ED represents not only a crisis but also a chance to lead a life more in tune with natural processes and physical well-being.

ED, by way of this cultural journey has developed into a concept and condition that not only describes a pathology but also offers a political critique of our contemporary societal condition. Although this development and the cultural tropes it makes use of are not entirely unique to ED, in comparison with other related diagnoses, this is a rather remarkable achievement for a medical condition that has existed for only 20 years. Certainly, this particular pattern of cultural tropes is only one possible way of experiencing and recovering from ED among many. But it is nevertheless an important and influential such pattern, a fact which is proved by the rising number of rehab- centres and methods focusing upon living in harmony with nature as a cure for ED. “Green rehab” is not unique to Sweden but it has become particularly influential in the country most probably due to the disorder of ED (Green rehab 2025a, 2025b, 2025c).

## The future of exhaustion disorder

In January 2024, a new report on ED was published by a group of researchers that partly overlap with the authors of the 2003 report discussed above: *Utmattningssyndrom*, main editor Åsberg (2024). In the new report, the authors survey the background and history of the diagnosis, make recommendations about how to perform the diagnostic procedure, and provide a scoping review of the research that has so far been carried out on the condition, including the results of various forms of treatment alternatives (mainly rehabilitation programs) so far. In the background overview of the report, they also make comparisons with how similar conditions have been diagnosed in other European countries.

The comparisons provide for very interesting reading, since the medical community and national institutions in Germany, France, The Netherlands, Belgium and the UK have not chosen to make a new diagnosis altogether, but rather continued to use the old concept of burnout sometimes with new specifications such as “clinical burnout” (Åsberg 2024, pp. 26–28). As mentioned above, the problem with using the burnout concept is that it is classified as a work-related problem rather than a medical diagnosis in ICD, but this has not stopped doctors and medical institutions in other countries than Sweden from listing it in medical charts and recommending sick leave and/or rehabilitation for the afflicted patients. As a matter of fact, the most studied group regarding burnout diagnoses in the world are the physicians themselves and, also, nurses, medical students and other health care staff.

The number of burnout diagnoses in the mentioned countries could be just as high or even higher than the number of ED diagnoses made in Sweden. However, this is very hard to know, since the concept of burnout is defined and made use of in a different way than ED and studied by way of different questionaries and scales in the various countries (Åsberg 2024, pp. 208–231). Also, it seems that the burnout concept in comparison with ED will more often lead to recommendations about changing the working routines rather than prescribing a long-term sick leave for the patient. Surprisingly, the burnout diagnosis therefore seems to provide for a quicker return to work than the diagnosis of ED, which, as we remember, was put in place to avoid stigma and associations of “having been burnt out” like a machine, or similar.

The new report also contains other disconcerting facts about the diagnostics of ED. Within the current medical practice of diagnosing and then attempting to treat the disorder, nothing in the treatment repertoire seems to help significantly beyond rest and sick leave in itself (also, doing absolutely nothing has a rather slow recovering effect, since the average recovery time is more than half a year). Psychotherapy by way of various methods (CBT, ACT and others), anti-depressive medication, rehab programs, including physical and psychological measures, nothing has been shown to have a statistically significant effect in comparison with no treatment in the case of ED (Lindsäter 2022; Åsberg 2024). This is bad news for the clinical utility of the diagnosis, but one has to keep in mind that a reason for failing to provide evidence for effective treatments could be the small number of studies carried out (29) and the limited number of possible patients in comparison to diagnoses that are being used worldwide.

The research group of the new report, which partly overlaps with the researchers that created the diagnosis in 2003 (Marie Åsberg being the editor in charge of both reports), suspects that the diagnosis of ED has been used too inclusively, not making sure that the patients suffer from the characteristic symptomology and properly distinguishing ED from depression, generalized anxiety disorder and acute stress disorder (Åsberg 2024, pp. 116–121). To make the diagnosis of ED more specific they propose new and sharpened diagnostic criteria compared to the existing ones.^7^

The main differences, compared to the 2003–2005 criteria, are that the period during which symptoms must have been present for a diagnosis to be made is increased from two weeks to a month and that increased stress sensitivity and cognitive disabilities are made into necessary criteria instead of belonging to a group of six symptom types out of which four must be present. The type of cognitive disabilities to be scrutinized are also specified in far more detail: “considerable difficulties to perpetuate, change, and direct attention. Memory problems. Difficulties to take in and process new information, sort and process impressions, solve problems and plan activities,” compared to the previous: “persistent complaints of impaired memory and concentration” (see above).

It is obvious that the leading researchers on ED in Sweden are concerned that the diagnosis is being overused and that the new proposed criteria should help to stop this development. This is rather startling, since the standard strategy of psychiatrists and medical researchers who have coined or helped to establish a new disorder has rather been to promote and expand them as far as possible (Horwitz 2021). The reason for the recommended turn towards a stricter symptomology of ED may be that the diagnosis is a Swedish outlier which no other country has accepted, but it may also mirror a new strategy among psychiatric researchers that is presently taking place in the realm of other exploding disorders, such as depression, bipolar disorder, ADHD and ASD (Rose 2019). The fear among the researchers is obviously that the medicalization process has gone too far, and that the dilution of psychiatric diagnoses makes them less respectable and trusted in the eyes of the public.

Meanwhile, the future of ED is far from obvious. The National Board of Health and Welfare in Sweden (Socialstyrelsen) may well follow the advice of the research group and change the criteria soon. If they do, patients will probably find it a harder to be diagnosed with ED and subsequently they will have a harder time to be granted with long time sick leave, since the recommendations made by The Swedish Social Insurance Company (Försäkringskassan) for alternative diagnoses, such as other stress disorders or depression, are not to stay home from work for several months (as it is for ED). Another possibility for The National Board of Health and Welfare would be to abandon the diagnosis altogether and instead recommend the use of the concept of burnout, maybe even specify it in the manner of “clinical burnout,” a development that has taken place in some other countries. This would make it easier to find evidence for different treatment options, including more specific rehab programs by way of country-to-country comparisons.

## Conclusion

To conclude, exhaustion disorder is a diagnosis that has been put in place and constituted by many different powers at play: changes of working life conditions, changes in health insurance regulations, proposals made by influential researchers, and, last but not least: changed labels and concepts in the heads of doctors and patients, thematized and disseminated by way of podcasts, novels and social media in popular culture. The genesis of ED in Sweden displays very clearly how diagnostic categories become lived and told by persons, who are integrated in a cultural matrix shared by patients, doctors and all other citizens of a country. It shows, in the words of the philosopher Ian Hacking, that psychiatric disorders are “interactive kinds” (Hacking 1999, pp. 103–105). According to Hacking, medical diagnoses, and particularly so in the case of psychiatric disorders, can be natural – residing in the brain – and culturally interactive at the same time, since patients become aware of and are affected by the labels they are classified with. The phenomenological analysis of illness suffering substantiates Hacking’s insight by showing how alienating experiences at the levels of embodiment, being-in-the-world and narrative patterns of personal identity relate to and influence each other by way of cognitive processes but also, and most significantly, by way of moods.

Cultural patterns established with the help of ED harbor possibilities for political critique of the situation of women in Western societies and also regarding the relations between human beings and other living creatures on earth. One interesting subspecies of ED-stories is a sort of feminist eco-critique, motivated by the identification of a medical diagnosis, which becomes reinterpreted by way of a narrative pattern. ED is portrayed as a consequence of living disconnected from nature and one’s bodily rhythms, becoming infected by the social pathologies of modern urban life, including the overuse of smart phones and other technical gadgets. Women are more susceptible to develop ED symptoms according to this view, yet they also possess unique pathways to reconnect with nature through their embodied experiences. In this way, an ED breakdown comes to represent not only a crisis but also a chance to change one’s life and become more in tune with natural processes and establish true physical well-being. The claim of this paper is not that this feminist eco-critique provides the only or even the dominant way to live with ED in Sweden today, but it is an influential cultural trope that has led to the establishment of many “green rehab” centers, and therefore worth thematizing in writing a first history of the disorder.

## Footnotes

1 Culture-bound syndromes, such as *taijin kyofusho* and eight others, are listed in DSM-5 as “cultural concepts of distress” (APA 2013, p.837). In ICD-11 it is mentioned under cultural aspects of social anxiety disorder, as a concept mainly made use of in Japan and Korea, with the code 6B04.

2 The number of sick-leave days in the Swedish statistics does not include the number of days that are compensated because of permanent or long-term disabilities. If these numbers were added to the days on sick leave, the average number of days in 2002 would have been 42 (ISF 2023, p. 33).

3 The reasons why sick leave days pro capita varies over time in a country are manifold. Some of the disparities are due to the spread of infectious diseases, influenza is far more prevalent in wintertime than in summertime, and the covid pandemic resulted in a peak of sick leave days during 2020, 2021 and 2022, for instance. Other variations depend on changes in the job market situation – when unemployment is high, people tend to not call in sick for work, since they are afraid to lose their jobs, a behavior that is often followed by a raise in the number of sick leave days when the job market turns better again (this is probably what happened in Sweden 1997). The rise in number of days on sick leave can also be the effect of more stressful working conditions, which tend to have more of a long-time effect on the health situation of employees (Johnson 2010). And last but not least, the numbers very much reflect the ever-changing rules of the health insurance system in a country and how these rules are attended to by doctors and administrative staff when granting sick leave or not. In Sweden, as we will see, all these factors need to be considered to make sense of the sizeable fluctuations in average number of days of sick leave from the 1990s and onward.

4 The details and results of the exploratory study referred to in the report remain unpublished until this day (Lindsäter 2022).

5 As a comparison, a search on PubMed on “exhaustion disorder” generates merely 45 scientific articles, all written by Swedish (or Nordic) authors.

6 The whole arcive of broadcasted programs is available at: https://sverigesradio.se/sommar-i-p1. I have not performed a comprehensive study of the entire list of hosts and programs since 2009, but my informed opinion would nevertheless be that the narrative pattern is present in at least 5 out of 60 programs every summer.

7 The proposed new criteria for ED are the following: A. The symptoms have developed in response to one or more significant and persistent stressors from which there has been offered no recovery or relief. The circumstances have been present for at least 6 months. B. A persistent feeling of severe exhaustion with significant lack of psychic energy since at least a month. C. Clinically significant cognitive disability since at least a month, including considerable difficulties to perpetuate, change, and direct attention. Memory problems. Difficulties to take in and process new information, sort and process impressions, solve problems and plan activities. D. Pronounced stress sensitivity since at least a month. Cognitive abilities are noticeably impaired by increased workload. E. At least three of the following symptoms have been present nearly every day during the last month: 1. insomnia or hypersomnia. 2. persistent complaints of physical fatigue or lack of endurance. 3. stimuli oversensitivity in response to normal conditions of light and sound. 4. physical symptoms such as muscular pain, chest pain, palpitations, nausea, gastrointestinal problems or vertigo. F. The symptoms cause clinically significant distress or impairment in social, occupational or other important areas of functioning. G. The symptoms are not due to the direct physiological effects of a substance (e.g. misuse of a drug or medication) or a general medical condition. H. The symptoms are not better explained by another psychiatric disorder or illness, or by a personality disorder, neurocognitive, neuropsychiatric or developmental disorder. (Åsberg 2024, p. 101, my translation)
